# The role of community health workers in prevention of blindness due to ROP

**Published:** 2018

**Authors:** Sai Kiranmayee P, Viswanath K, Muralidhar O

**Affiliations:** Consultant: Vitreo-Retinal Services, Pushpagiri Vitreo-Retinal Institute, Secunderabad, India.; Chairman: Pushpagiri Vitreo-Retinal Institute, Secunderabad, India.; Director: Pushpagiri Vitreo-Retinal Institute, Secunderabad, India.

**Figure:**
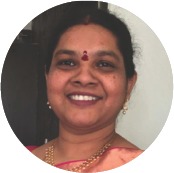
Sai Kiranmayee P

**Figure:**
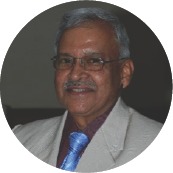
Viswanath K

**Figure:**
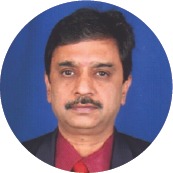
Muralidhar O

**Community health workers can play an important role in health education and ante-natal care that will help reduce the number of preterm births. As members of their communities, they are best placed to advise and support parents and extended families in reducing the risk of ROP and visual loss.**

**Figure:**
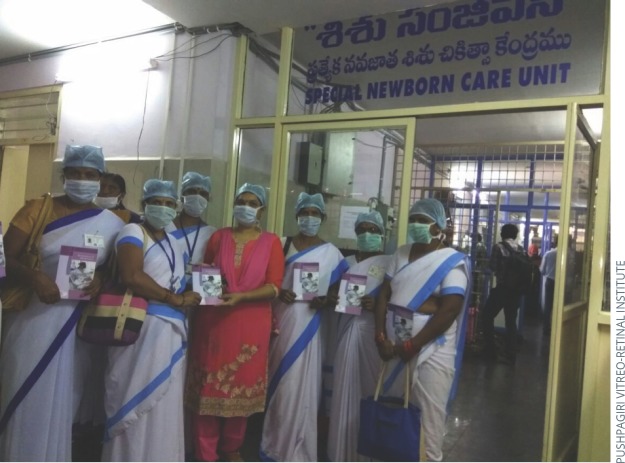
ASHA workers visit a Special Newborn Care Unit (SNCU). INDIA

Preterm births are an important public health concern worldwide due to the resultant morbidities. Survival of premature babies has improved largely due to improved neonatal care services. However premature infants are at greater risk of cerebral palsy, developmental delays, hearing and vision related issues.

Retinopathy of prematurity (ROP) remains one of the leading causes of childhood blindness worldwide. Recent estimates show that worldwide 32,000 infants become blind or visually impaired due to ROP. Most of the ROP blind infants are born in countries in Asia.^1^ Although the numbers seem insignificant, the number of blind years is huge, leading to an immense socio-economic burden to the family, society and country. Currently, ROP is a significant problem even in district and remote hospitals in rural India.^2^ Community-based interventions help in reducing perinatal and neonatal morbidity and mortality.^3^ The most promising short-term strategy for providing newborn care entails training and equipping community health workers.

## Community health workers in India: role in ROP programmes

Community health workers are members of communities where they provide preventive, promotional and rehabilitation care to other members. The community health workers in India are

- Auxiliary nurse midwife (ANM)
- *Anganwadi* worker (AWW) and
- Accredited social health activist (ASHA worker).

They play a crucial role in the health care system especially in maternal and child health care and thus can help in prevention of blindness due to ROP.

### Auxiliary nurse midwife

Commonly known as ANM, they are a village level female health worker who is the first contact person between community and health services. ANMs are regarded as the grassroots workers in health organisation pyramid.

### *Anganwadi* Workers

*Anganwadi* centers are government run mother and child care center in villages in India. The anganwadi workers ensure antenatal and postnatal care for pregnant women, nursing mothers and immediate diagnosis and care for new born children. Monitoring regular health and medical check-ups for women and children is one of their key responsibilities.

### Accredited social health activists

ASHA workers are local women trained to act as health educators and promoters in their communities. Their tasks include motivating women to give birth in hospitals, bringing children to immunisation clinics, encouraging family planning, treating basic illness and injury with first aid, keeping demographic records and improving sanitation.

Community health workers serve as a key communication pathway between the healthcare system and the rural population, especially in reducing preterm deliveries and in prevention of ROP in babies born too soon. Preterm deliveries can be reduced by working on modifiable risk factors such as maternal nutrition, pregnancy planning, birth spacing etc. ROP blindness can be reduced by expanding and improving screening and treatment services at medical colleges and district hospital sick newborn care units (SNCUs) and neonatal intensive care units (NICUs). The community health workers play a key role in preventing ROP blindness in three different stages:

- Reducing preterm deliveries
- Prevention of ROP in preterm babies
- Screening and treatment services for ROP

## Role of community health workers in prevention of ROP blindness

### 1. Reducing preterm deliveries

#### a. Child marriages and early pregnancy

Child marriage has lasting consequences on girls, from their health, education and social development perspectives which often last well beyond adolescence. It has been found that teenage mothers are three times more likely to deliver preterm babies and twice as likely to deliver low birth weight (LBW) babies compared to older mothers (21 Yrs to 34 Yrs).^4^ Health workers, especially in rural areas can counsel newly weds and public about such age-old harmful practices.

#### b. Birth spacing

It is likely that when the space between births is short, there will be depleted nutrition in new mothers, as these women do not get enough time to recover before getting pregnant again. Therefore, after a birth, the interval before attempting a new pregnancy should be at least 24 months to reduce the risk of adverse maternal and infant outcomes.^5^ Community health workers can explain that optimally spaced births reduce the infant and maternal morbidity and mortality. They can help in educating their communities about different family planning methods and encourage their use for optimal birth spacing.

#### c. Maternal nutrition

Maternal undernutrition is still a major problem in India. In populations with food insecurity and high rates of maternal undernutrition, balanced protein energy supplementation may improve foetal growth and reduce the risk of foetal and neonatal death.^6^ Community health workers can monitor and advise on proper dietary intake of balanced energy and protein contents. As anaemia is more common in rural women, iron supplements may be provided to them. The health workers may provide periconceptual folic acid supplements which help in reducing neural tube defects, preterm births and low birth weight. As village health workers are trusted by friendly pregnant women, they can monitor and guide them about their diet and nutritional supplements from time to time.

#### d. Stress during pregnancy

Prenatal maternal stress, depression and anxiety are found to be related to preterm labour.^7^,^8^ A community health worker can explain to the spouse of the pregnant woman and their family members about the ill effects of domestic violence on the outcome of pregnancy.

#### e. Antenatal check-ups and hospital deliveries

ASHA workers can help in educating pregnant women about regular antenatal check-ups and the importance of periodic follow-up. This aids in monitoring for hypertension, diabetes, infections etc, which if properly managed may reduce preterm deliveries. The community health workers play a role in birth preparedness which consists of preparing the mother, family and community for delivery and potential complications. They should encourage and increase the percentage of hospital deliveries which are safe for the mother and child.

**Figure 1:**
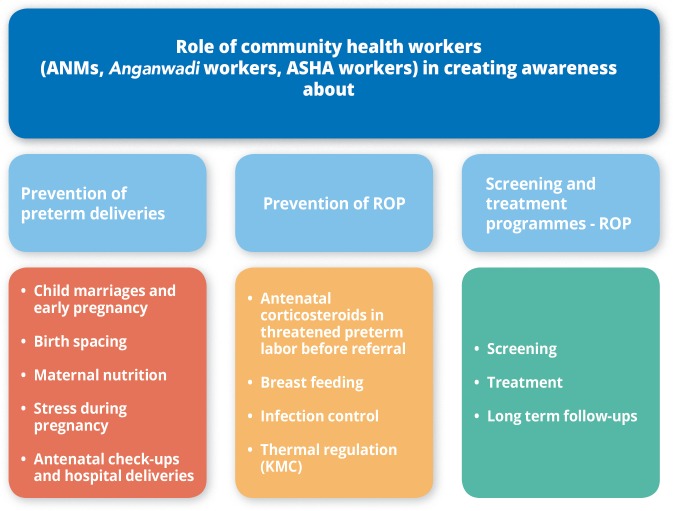
Role of community health workers

### 2. Prevention of ROP in a preterm infant

With a preterm birth, the focus shifts to prevention of ROP and other morbidities. A well- trained health worker anticipates and prepares to minimise the morbidities that may ensue. Risk factors like poor weight gain, infection etc if tackled properly can prevent ROP related childhood blindness.

#### a. Antenatal corticosteroids (ANC) in threatened preterm labour

A significant reduction in the risks of mortality, respiratory distress syndrome and intraventricular haemorrhage have been confirmed after administering antenatal steroids in babies delivered before before 34 weeks gestation.^9^ ANMs who are skilled birth assistants should be guided on giving ANC to pregnant women in preterm labour. Guidelines include diagnosis of preterm labour, indications, contraindications and doses of ANC. Thus, the health workers help in prevention of ROP by giving prereferral dose of steroid and arrange for referral to an appropriate facility.

**Figure:**
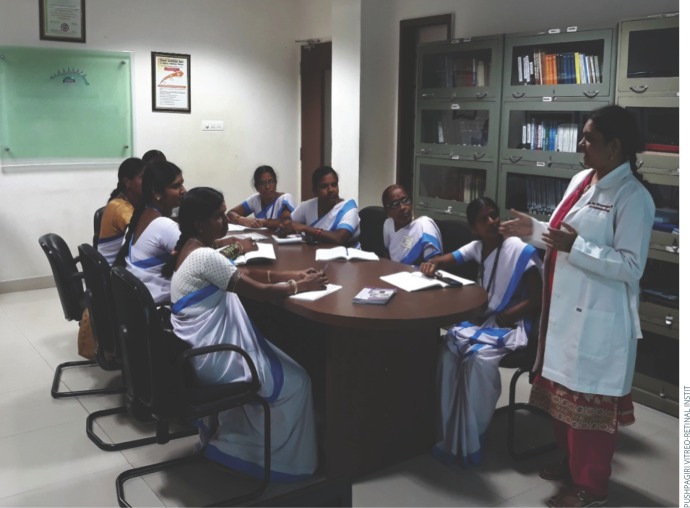
Sensitisation of ASHA workers. INDIA

#### b. Breastfeeding

It has been found that exclusive breastfeeding for six months starting within an hour after birth may prevent ROP. Health workers can help in explaining to mothers the importance and advantages of exclusive breast feeding. There are several significant short term and long term benefits of breastfeeding preterm infants. Neurodevelopmental outcomes are also proven to improve with early and exclusive breastfeeding.^10^ The health worker can also be trained to check for proper weight gain.

#### c. Infection control

As sepsis in preterm infants leads to increased risk of ROP, infection control procedures like personal hygiene can be clearly explained to mothers. Small yet significant measures such as bathing regularly and washing all clothes used for the baby can be easily explained to the mothers in the local language. Educating family members along with the mother on washing hands thoroughly before touching the baby and keeping surroundings clean help in keeping infections under control.

#### d. Thermal care

Hypothermia is another concern in the management of pre-term infants. Kangaroo mother care (KMC) involves direct and continuous skin to skin contact between infant and mother. It helps in preventing hypothermia, improving weight gain and reducing incidence of infection. The procedure and benefits of KMC when explained clearly to the mothers, help in improving survival and decrease ROP. Along with KMC, the ASHA workers can also show proper swaddling of babies and keeping babies warm.

### 3. Screening and treatment programmes for ROP

#### a. Screening for ROP

Community health workers should be made aware that all the babies born too soon (<34 weeks) and small (<2000grams) who have been admitted to SNCU/NICU should undergo first eye screening for ROP before 30 days of birth. They are best placed to motivate parents to take their preterm infant for ROP eye screening to a trained ophthalmologist nearest to them. Educating parents about the disease with the help of visual aids like flash cards or posters of the disease and how it leads to blindness helps in reaching out to illiterate population in a better way. Health workers can also encourage parents to take the baby for ROP screening before they are 30 days old.

#### b. Treatment

Community health workers can help parents understand that ROP is a disease with a narrow time period between detection and treatment and that treatment cannot be delayed. Counselling parents and their families about laser photocoagulation can also be done by a community health worker.

#### c. Long term follow-up

The most common challenge faced in management of ROP is lack of compliance and follow-up. Though the initial screening is done when the baby is in NICU, parents especially in rural areas do not come back for follow-up. As ROP is a disease which requires multiple visits the ASHA workers can help in tracking pre-term infants in their communities and motivating parents to go for follow up visits. Educating parents about long term effects a pre-term birth can have on the eyes is also important, to encourage them to attend for follow up. Simple language must be used in training the ASHA workers so they can effectively counsel parents.

**Figure:**
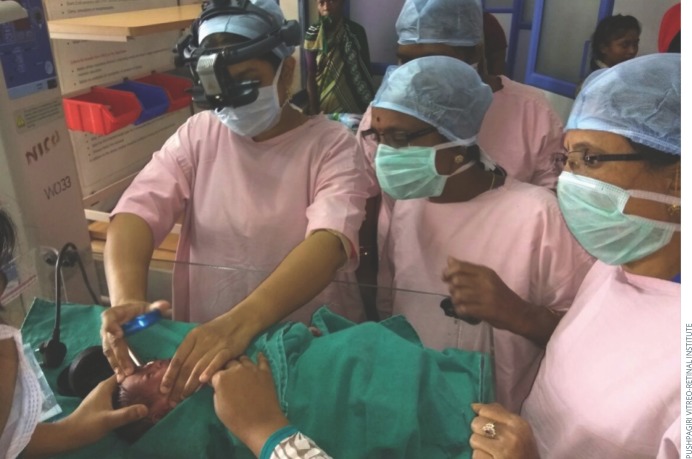
ASHA workers observing ROP screening. INDIA

## Conclusion

Community health workers can play an important role in health education and ante-natal care that will reduce the number of premature babies born. They also have an important role in advising and supporting the mothers of preterm babies in order to reduce the risk of ROP and visual loss.
